# Transcranial direct current stimulation for the treatment of post-stroke depression: A systematic review

**DOI:** 10.3389/fneur.2022.955209

**Published:** 2023-01-18

**Authors:** Wenjian Hao, Yong Liu, Yuling Gao, Xiaoyang Gong, Yi Ning

**Affiliations:** ^1^Department of Rehabilitation Medicine, The First Affiliated Hospital of Dalian Medical University, Dalian, China; ^2^Institute (College) of Integrative Medicine, Dalian Medical University, Dalian, China

**Keywords:** stroke, depression, mechanism, transcranial direct current stimulation, post-stroke depression (PSD)

## Abstract

**Background:**

Post-stroke depression (PSD) is not only a frequent neuropsychiatric manifestation secondary to stroke but is also associated with disability, poor rehabilitation outcomes, sleep disorders, cognitive impairment, and increased mortality. Transcranial direct current stimulation (tDCS), a primary modality of non-invasive brain stimulation (NIBS), has shown promising clinical results in the rehabilitation of patients with PSD recently. The primary aim of this systematic review is to assess the effects of tDCS on PSD.

**Methods:**

PubMed and Cochrane databases were used for paper identification up to May 2022. Only English language studies and published data were taken into consideration. The methodological quality of selected studies was assessed according to the modified Sackett Scale, based on Physiotherapy Evidence Database (PEDro) scores.

**Results:**

Six experimental studies were included for the PSD treatment of tDCS and all of them reported that, following the intervention of tDCS, the experimental group shows a statistically significant decrease in the depression level in accordance with different assessment scales.

**Conclusion:**

This article simply aims at providing a comprehensive overview of the raw data reported in this field to date. Based on the current evidence, tDCS presents promising results for the treatment of PSD. Moreover, tDCS is also effective in PSD patients with aphasia or CPSP. However, an optimal stimulation protocol is needed to formulate. Thus, the development of robustly controlled, randomized, and high-quality clinical trials to further assess the utility of tDCS as a therapeutic tool for the treatment of PSD survivors is encouraged.

**Systematic review registration:**

https://www.crd.york.ac.uk/prospero/display_record.php?ID=CRD42023322076, identifier: CRD42023322076.

## Introduction

Stroke is a cerebrovascular disease with a high incidence worldwide. It is mainly manifested as a series of pathological reactions caused by ischemic or hemorrhagic injury of the brain tissue. Among the complications of a stroke, post-stroke depression (PSD), a source of suffering among stroke survivors (1), is the most frequent psychiatric problem. Persons with PSD are strongly associated with higher mortality rates (2, 3), higher rates of suicidal ideation (4), and lower quality of life compared with post-stroke patients without depression. Hence, it is vital to have knowledge of the principles of identification and effective treatment options for PSD.

The pathophysiology of PSD is complicated and still incompletely understood, which may include a result of the joint action of multiple mechanisms. One of the most widely accepted hypotheses is the monoamine neurotransmitter hypothesis, represented by low levels of expression. Other processes that may contribute to PSD include the reduction of brain-derived neurotrophic factor (BDNF) content, excess of inflammatory cytokines, dysfunction of the hypothalamic-pituitary-adrenal axis, neuroanatomical mechanism, and glutamate-mediated excitotoxicity (5–7). A recent study suggests that the gut microbiome may play a role in the development of PSD (8), which may be involved in the regulation of lipid metabolism.

The main symptoms of PSD include persistent low mood, lack of interest, apathy, slow thinking, pessimism, and even suicidal thoughts. The fifth US Diagnostic and Statistical Manual of Mental Disorders (DSM-5) is currently the most commonly used scale to diagnose PSD. However, when used in busy and resource-poor clinical settings, the DSM-5 may not be validated for use in stroke. As a result, it is often appropriate to use a self-completed depression screening scale, such as the 9-item Patient Health Questionnaire, the Hamilton Depression Rating Scale (HDRS), the Beck Depression Inventory (BDI), Hospital Anxiety and Depression Scale (HADS), and the Montgomery-Åsberg Depression Rating Scale (MADRS) (9).

With the standardized use of new antidepressants and the rapid development of psychotherapy pharmacological treatment (10), psychosocial interventions (11), traditional Chinese medicine (TCM), especially non-invasive Brain Stimulation (NIBS) technology, and the quality of life of patients with PSD has improved dramatically. Pharmacotherapy is still the first-line treatment of PSD with an improvement of cognitive impairment and long-term survival (1, 12), although with a controversial efficacy (13), frequently accompanied by a high risk of adverse outcomes (14). TCM may be a potential selection for patients with PSD who fail to afford high charges of psychotherapy or other PSD treatments or are unable to tolerate antidepressant side effects. Acupuncture, an effective form of practice of TCM, is a promising effective therapy that is gradually being accepted as a therapeutic option for neuropsychiatric disorders across the world (13, 15, 16).

Recently, the role of NIBS in the rehabilitation of cognitive impairments after stroke has attracted much attention (17). The main modalities of NIBS are repetitive transcranial magnetic stimulation (rTMS) and transcranial direct current stimulation (tDCS), which are emerging neuromodulation techniques that are beneficial to the recovery of dysfunction after stroke (18–21).

Transcranial direct current stimulation is mainly used to regulate the cortical excitability under the stimulated brain regions (22) through constant and low-intensity current (0.5–2.0 mA), which can effectively change the polarization state of the cell membrane and modulate the plasticity of synapses (23). The anode electrode is usually applied to area C3 or C4, while the cathode electrode is mostly positioned on the contralateral supraorbital area (24, 25). C3 or C4 is the reflex region of the primary motor cortex according to the 10–20 electroencephalography [EEG] system. Anode tDCS stimulation can increase stimulated cortical excitability, while cathode stimulation decreases it (23).

Transcranial direct current stimulation is currently widely used in neuropsychiatric disorders, such as depression, post-stroke aphasia, and Parkinson's disease (26). However, the neurobiological mechanisms underlying tDCS remain elusive, involving several pathological processes in the central nervous system, such as modulating the resting membrane potential of the targeted neuronal population (27), enhancing the functional connectivity between two brain regions (28, 29) and increase the synaptic plasticity, which can be achieved by inducing the release of neurotransmitters, modifying the activity of N-methyl D-aspartate (NMDA) receptor (30, 31) and inducing the occurrence of long-term potentiation (LTP) of cortical recombination (32). BDNF, which plays a key role in LTP formation, is modulated by tDCS based on some studies (33). TDCS also has long-lasting after effects (34). Research confirms that after 5 min tDCS anode stimulation, it can induce increased excitability of the motor cortex, which lasts for more than a few minutes (35). TDCS after effects is affected by a lot of factors, such as duration and frequency of stimulation, locations of anode/cathode electrode, current density, and co-administered treatments (31).

In view of the increasingly obvious disadvantages of various treatment methods for PSD, tDCS, as a novel treatment method, has attracted more and more scholars' attention. But the effectiveness is not yet well established. Therefore, we conducted a systematic review of the clinical studies on tDCS in the treatment of PSD in recent years to contribute to the standardized use of tDCS and improve the wellbeing of patients with PSD.

## Methods

This systematic review was registered in the International Prospective Register of Systematic Reviews (PROSPERO) database (CRD42023322076). The review was administrated in accordance with PRISMA (Preferred Reporting Items for Systematic Reviews and Meta-Analyses) guidelines.

### Search strategies

The study search was to capture as many relevant clinical studies as possible. This article referred only to published data. PubMed and Cochrane databases were used for paper identification up to May 2022. Only English language studies and published data were taken into consideration. The search strategies combined medical subject heading (MeSH) with free-text terms, which were adjusted in terms of the requirements of a specific database. Our key search terms were stroke, depression, and transcranial direct current stimulation.

Two authors (WJH and YN) managed the literature searches and strictly screened eligible research according to inclusion and exclusion criteria. When it came to any disagreements, a third author (YL) was consulted to cope with inconsistencies. Two authors (WJH and XYG) were assigned to carry out the data extraction. Subsequently, all the authors assessed the methodological quality of each article and then crosschecked it to ensure accuracy. The search strings used in both databases are shown as Supplementary material.

### Inclusion and exclusion criteria

Selected studies had to meet the following inclusion criteria: (a) the main intervention was tDCS; (b) the primary subject of the study is people; (c) the principal diagnosis for patients was PSD [patients diagnosed with stroke with brain neuroimaging, clinical history, and physical examination; diagnosis with depression mainly according to the Mini-International Neuropsychiatry Interview (MINI) questionnaire, the Beck Depression Inventory (BDI)], DSM-IV, or DSM-V; (d) 10 is the minimal amount of tDCS sessions; (e) Published in English; and (f) Peer-reviewed.

The reasons for excluding studies were as follows: (a) Relevant indexes were not reported; (b) Studies with the main diagnosis were anxiety, epilepsy, or other cognitive disorders; (c) Duplicate publications; and (d) Valid data were unavailable or data not completed.

### Data extraction

According to the PRISMA guidelines, we used the PICOS tool, which was more sensitive than other search tools such as SPIDER or PICO, and was recommended for current practice to ensure exhaustive literature searches for our research (36, 37). We paid particular attention to patients' features (gender, age, sample size, post-stroke time onset, diagnosis and diagnosis instruments, stroke type, and lesion position), intervention, machine type, comparator, outcomes, study design, and stimulation parameters. Then, we analyzed the similarities and differences among the selected articles. The specific PICOS model is shown in Table 1.

**Table 1:** PICOS model.

P-Participants	Adults (>18 years) Patients with primary diagnosis of Post-stroke depression(PSD)
I-Intervention	Patients with PSD mainly treated with transcranial direct current stimulation (tDCS)
C-Comparator	A control group comparable to the experimental group
O-Outcomes	Scales mainly used to assess depression
S-Study design	All design studies

PICOS, Patient, Intervention, Comparator, Outcome, study design.

### Study quality assessments

The methodological quality of selected studies was assessed according to the modified Sackett Scale, based on Physiotherapy Evidence Database (PEDro) scores (38). The PEDro has 11 items on study quality, each of the concepts answered with “yes” (score = 1) or “no” (score = 0). The PEDro has been shown to be a more comprehensive measure of the methodological quality for trials in the stroke rehabilitation literature compared with others such as the Jaded scale.

## Results

**Figure 1:**
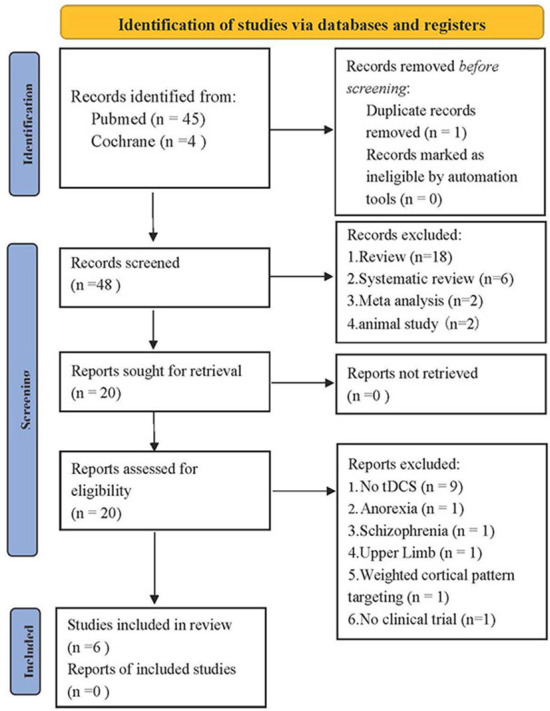
PRISMA 2020 flow diagram for new systematic reviews which included searches of databases and registers (39).

From our literature search, 49 records were identified through databases. Besides 1 duplicate removed, 28 articles were excluded based on their titles and abstracts, and 14 articles were excluded owing to inconsistency with inclusion criteria. A total of three types of clinical research were screened in terms of the PICOS rule. However, due to the small number of selected research, three case reports were also included to fully discuss the research status of tDCS application in PSD. After the full-text assessment, six studies were included in this systematic review. Figure 1 is the flow diagram of the study selection process.

### Quality assessments

Six experimental studies of PSD were included in this systematic review (40–45) with tDCS treatment. The methodological quality of Valiengo's randomized, placebo-controlled clinical trial (RCT) was high, with a mean PEDro score of 8 out of 10 (level 1b evidence). Both Li et al. and An et al. had a control group and were rated as level 3 on the modified Sackett Scale. The case series study (42) and the case reports (41, 45) were considered as level 4 and 5 evidence, respectively.

### Participants' characteristics

A total of 120 patients diagnosed with PSD were involved in this study. Following is a detailed description of homogeneous characteristics: (a) All the patients with PSD were diagnosed by physical examination, neuroimaging, and scales, specifically made for assessing the depression level. (b) Participants' age ranged between 32 and 74 years. (c) The time for stroke varies between 2 and 24 months. (d) Stroke types included hemorrhagic and ischemic stroke. Lesion position is particularly presented in Table 2. (e) All patients were antidepressant-free across six studies.

**Table 2:** Summary of the participant's characteristics in the active/experimental group.

**Authors**	**Sample (*n*)**	**Diagnosis and diagnosis instruments**	**Female**	**Male**	**Mean age(SD)**	**Time from stroke, months**	**Stroke type and lesion position**
Bueno et al. (41)	1	PSD M.I.N.I. question-naire	1	0	48	3	Ischemic stroke: the left basal ganglia and left insula.
Valiengo et al. (43)	48 (5 dropped out), 24 in the active group	PSD Stroke:brain CT or MRI; Depression:MINI,DSM-IV	12	12	62.2 ± 12.3	11.1 ± 2	Right side (stroke) 11; subcortical structures (stroke) 10; frontal injury (stroke) 8; ischaemic stroke 21.
Valiengo et al. (42)	4	PSD with aphasia Stroke: brain neuroimaging, clinical history, and physical examination; Depression: MINI questionnaire; Broca's aphasia:a certified speech-language pathologist	4	0	48.25 ± 11.61	6 ± 4.08	Hemorrhagic 1; ischaemic 3.
An et al. (40)	40 20 in the experimental group	PSD Stroke:CT or MRI; Depression:BDI scores>16.	3	17	51.0 ± 11.7	14.6 ± 6.3	Cerebral infarction 11, cerebral hemorrhage 9; Left paralysis 13, right paralysis 7.
Li et al. (44)	26 (4 dropped out) 12 in the experimental group	PSD DSM-5	6	16	55.67 ± 9.07	3.13 ± 1.45	Hemorrhagic 7; ischemic 5.
Hassan et al. (45)	1	PSD and CPSP BDI and DN4Q respectively	1	0	45	4	Ischemic stroke.

BDI, Beck Depression Index; CT, computed tomography; DN4Q, Douleur Neuropathique 4 Questionnaire; DSM-IV, the Diagnostic and Statistical Manual Fourth Edition; DSM-5, the Diagnostic and Statistical Manual of Mental Disorders, Fifth Edition; M.I.N.I., Mini International Neuropsychiatric Interview; MRI, magnetic resonance imaging.

### Stimulation protocols

In five studies (40–44), the anode electrode was placed on the scalp corresponding to the left DLPFC, while the cathode was attached to the right DLPFC according to the International 10–20 EEG System. However, in Hassan's study, the anode electrode was placed over the right DLPFC and the cathode on the contralateral supraorbital region.

Transcranial direct current stimulation was delivered at an intensity of 2 mA (current density = 0.80 A/m^2^) for 30 min in the active/experimental group in four studies (40–43), while in Li and Hassan's studies, the patients received anodal tDCS stimulation at an intensity of 2 mA for only 20 min.

The number of sessions in Valiengo's two studies is 12, comprising once daily on weekdays for 2 weeks as well as two additional sessions after 2 and 4 weeks. However, in Bueno and Hassan's studies, patients received only 10 sessions in contrast to the 20 sessions that patients received in An and Li's studies. In three studies (40, 43, 44), the stimulation was stopped at 15, 30, and 60 s after the application in the control/sham group, respectively, though the anode and cathode positions were the same as in the active/experimental group. A specific description is presented in Table 3.

**Table 3:** Summary of tDCS study characteristics.

**Aims**	**Study design**	**Author**	**Research Institute**	**Anode**	**Cathode**	**Current density (A/m^2^)**	**Number of sessions**	**Machine type**	**Concomitant therapy/tasks**	**Results**	**Assessments**	**Limitations**
Mood and cognitive effects show the feasibility and initial response in a patient with PSD refractory to antidepressants to encourage further randomized clinical trials.	an open label case report	Bueno et al. (41)	None	the left DLPFC,F3	the right DLPFC	2 mA,30min	10	None	Fluoxetine dose	a significant mood and neurocognitive improvement	HDRS MADRS BDI MMSE MOCA Rankin	Not mentioned.
To assess the efficacy and safety of tDCS.	a randomized, sham-controlled, double-blind trial design	Valiengo et al. (43)	the University Hospital, University of São Paulo, São Paulo,Brazil	the left DLPFC,F3	the right DLPFC	2 mA,30min	12	DC-Stimulator, Neuroconn, Ilmenau, Germany	None	Active tDCS was superior to sham at end point. Response and remission rates were higher in the active (37.5% and 20.8%, respectively) vs. the sham group.	The HDRS-17 score,clinical response, remission, the MADRS, the Rankin scale and the Barthel index, a tDCS adverse effects questionnaire, the Young Mania Rating Scale	1.Not performing MRI scans in all patients at baseline; 2.Not simulating the current distribution in computer models; 3.Small sample size.
To investigate the safety and the efficacy of a novel form of tDCS as therapeutic treatment of PSD in aphasic patients.	an open-label and uncontrolled design	Valiengo et al. (42)	the Local and National Ethics Committee of the University Hospital of the University of São Paulo	the left DLPFC,F3	the right DLPFC	2 mA,30min	12	None	None	A decrease in SADQ (47.5%) and in ADRS (65.7%) and the improvement was maintained four weeks after the treatment.	ADRS,a questionnaire for the evaluation of tDCS adverse effects	1.Having not formerly investigated the potential changes in language deficits; 2.Using an open-label and uncontrolled design.
To assess the effects of transcranial direct current stimulation (tDCS) on depression and quality of life (QOL) in patients with stroke.	controlled	An et al. (40)	the M rehabilitation center in Busan	the left DLPFC	the right DLPFC	2 mA,30min	20	Phoresor^®^ PM 850 (Phoresor^®^ II Auto Model No. PM 850, IOMED, Inc., Salt Lake City, USA)	Conventional occupational therapy	a significant decrease in depression and an increase in the QOL	The BDI and the SS-QOL	1.Small sample size; 2.other factors influencing QOL have not been investigated.
To investigate the neural mechanism of tDCS in the treatment of PSD using fNIRS.	controlled	Li et al. (44)	the Department of Neurorehabilitation at the China Rehabilitation Research Center	the left DLPFC,F3	the right DLPFC,F4	2 mA,20 min	20	Jiangxi Jingxin Medical Technology Co., Ltd., JX-tDCS-1	Drug treatment (sertraline hydrochloride 50 mg qd)	Reaction times during the working memory task were shorter (*P* < 0.05) and relative Oxy-Hb concentration changes were higher (*P* < 0.05).	An emotional face sex judgment task and a ‘1-back’ working memory task (before and after the treatment).	Using antidepressants and no follow-up of patients.
To share the effectiveness of using tDCS of the DLPFC with short inter-session intervals to reduce central pain and depression in a stroke survivor.	a case report	Hassan et al. (45)	the Physiotherapy Department of the Federal Medical Center, Nguru, Yobe State, Nigeria	the left DLPFC	the right DLPFC	2 mA,20 min	10	None	None	Following the application of the second protocol of stimulation, the BDI score improved while the pain(both VAS and DN4Q) became completely abolished.	the VAS, the DN4Q, the BDI	1. Not assessing the patient' improvement of her activities of daily living using any appropriate instrument; 2. Not checking whether the stimulation of both DLPFC and M1 will provide better outcomes.

ADRS, Aphasic Depression Rating Scale; BDI, the Beck Depression Index; CPSP, central post-stroke pain; DLPFC, dorsolateral prefrontal cortex; DN4Q, Doueleur Neuropathique 4 Questionnaire; fNIRS, functional near-infrared spectroscopy; MADRS, Montgomery-Åsberg Depression Rating Scale; M.I.N.I., Mini International Neuropsychiatric Interview; MMSE, Mini Mental State Examination; MOCA, Montreal Cognitive Assessment; PFC, prefrontal cortex; QOL, quality of life; SS-QOL, stroke-specific quality of life; VAS, visual analog pain scale.

### Concomitant therapy/tasks

In Bueno and Li's studies, patients were treated with antidepressants (fluoxetine dose and sertraline hydrochloride of 50 mg qd, respectively) during tDCS stimulation. While in An's studies, conventional occupational therapy was used as the concomitant task. No other intervention or pharmacological treatment was mentioned in Hassan and Valiengo's studies (42, 43, 45) except for tDCS.

### Placebo

Only in Valiengo's study in 2017, the authors used a randomized, sham-controlled, and double-blind trial design with the sham group consisting of only 60 s of stimulation.

### Depression

Five scales were used to investigate the depression level of PSD across the six studies. HDRS was applied in two studies. In one study, HDRS was one of the moods and cognitive rating scales (41); in the other study, differently, HDRS-17 (the 17-item version) was the primary outcome (43). The MADRS has also been used in Bueno's and Valiengo's (43) studies. The BDI was administered to score the depression levels in patients before and after the intervention in Bueno's, Hassan's, and An's studies.

Two of the five scales were specific for aphasic patients with PSD in Valiengo et al.'s study (42). A 9-item interview, the Aphasic Depression Rating Scale (ADRS), used as the primary outcome in Valiengo et al.'s study (42), was executed at the baseline (before the tDCS treatment), Week 2, Week 4, and Week 6 to evaluate depression degree in patients with aphasia. In addition, the Stroke Aphasic Depression Questionnaire (SADQ), consisting of a 21-item questionnaire, was applied in Valiengo's study to detect low mood in stroke patients with aphasia.

### Safety and adverse effects

Two studies (42, 43) assessed the safety with a tDCS adverse effects questionnaire, and both of them showed no adverse effects were observed. There were no side effects were reported in other included studies, and the treatment was well tolerated.

### Outcome

Bueno et al. first analyzed the feasibility of tDCS in the treatment of patients with PSD in 2011. In this open-label case report, a 48-year-old woman, who was diagnosed with PSD, showed marked amelioration of significant mood and cognitive impairment in the HDRS, BDI, MADRS, MOCA, and MMSE following the combination of anodal stimulation over the left DLPFC with fluoxetine dose. These positive results were intended to encourage further controlled trials on the field. Subsequently, in 2017, Valiengo et al. first conducted a randomized, sham-controlled study to verify that tDCS was effective and safe for PSD. Prior to this, a preliminary, open-label study was conducted by Valiengo et al. (42) to assess the safety and efficacy of tDCS for PSD patients with aphasia. In this study, four drug-free female patients with PSD who, due to their aphasia, showed improvement in depression after 12 sessions as manifested by a decrease in the Aphasic Depression Rating Scale (ADRS). Moreover, this improvement was maintained for 4 weeks after the treatment.

One year later, in Valiengo's controlled trial, 48 antidepressant-free patients with PSD met the inclusion criteria, and 43 completed the study (five patients dropped out). With the similar stimulation protocol described in Valiengo et al.'s study (42), the active group showed greater improvement in depressive symptoms as shown in HDRS-17 and also presented higher response (categorical, defined as ≥50% reduction from the baseline HDRS score) and remission (categorical, defined as an endpoint HDRS score of <8) rates in the active vs. the sham group. The authors recommended that tDCS was a favorable and safe option for PSD.

To assess the effects of tDCS on depression and quality of life (QOL) in patients with stroke, 40 patients were confirmed to be severely depressed and completed the experiment in An's controlled study. The BDI was administered to score the depression levels in patients before and after the intervention. They drew the conclusion that tDCS intervention caused improvement in depression levels as well as QOL in the experimental group, which might introduce a new outcome measure for the evaluation of the efficacy of tDCS in the treatment of PSD. However, the small sample size limited the generalization of the positive result.

Li et al. used fNIRS to investigate the neural mechanism of tDCS in the treatment of PSD. With the semblance stimulation protocol described in Valiengo et al.'s study (42, 43), two tasks (an emotional face sex judgment task and a “1-back” working memory task) were arranged for 26 patients with PSD to evaluate reaction times and relative concentration changes of oxyhemoglobin (Oxy-Hb) in the prefrontal cortex (PFC). As shown in the result, there was no notable difference between the experimental and the control group in the first task. In the second task, there was a statistical difference between the two groups with shorter reaction times in the experiment group. As for relative Oxy-Hb, both the left and right PFC in the experimental group showed a significant result. This study provided insight into the mechanism by which tDCS improves patients with PSD, enhancing aerobic metabolism in the PFC.

Hassan et al. shared the latest case report about the effectiveness of using tDCS over the DLPFC with short inter-session intervals to reduce central pain and depression in a stroke survivor who presented with central post-stroke pain (CPSP) and depression, following a stroke. The BDI score declined from 25 to 7 after the intervention of 2 mA, and 20 min of anodal tDCS stimulations for 2 weeks. However, the BDI score returned to 25 at 3 weeks post-intervention. After the second protocol of stimulation (seven daily sessions of stimulations of 2 mA, 13 min, each with 20 min inter-session intervals for 1 week), the pain score turned to 0 immediately, while the BDI score improved to 18 at 3 weeks and later to 7 at 6 months post-intervention, which might be related to a higher tDCS dose and the aftereffects of tDCS.

## Discussion

Post-stroke depression is a common neuropsychiatric complication after stroke (46). Although the pathophysiology or pathogenesis of PSD is complex and largely unknown, there are increasing treatments such as pharmacotherapy, psychotherapy, psychosocial–behavioral intervention, TCM, and especially NIBS to deal with PSD. In this review, we have included six studies to provide a relatively comprehensive summary of the raw data reported in this area so far. Although all the studies show marked improvement of depressive symptoms in patients with PSD, there are some questions needed to be further explored.

Biomarkers reflecting the effects of tDCS and predictors of PSD need to be further explored. In addition, there is no universal standard for the diagnosis of PSD. Therefore, it is difficult to make a comparison between different studies because the six included studies hold different diagnostic criteria for PSD.

Transcranial direct current stimulation seems to show a promising result in the treatment of patients with PSD and with aphasia according to Valiengo et al.'s study in 2016. Aphasia can be a frequent complication following a stroke. However, with a missing investigation of the potential changes in language deficits before the study, this study cannot provide strong evidence that tDCS is effective for aphasia. With a growing number of evidence supporting the enhancing effects of tDCS in the recovery of post-stroke aphasia (47, 48), controlled and randomized clinical trials to further verify the utility of tDCS for the treatment of PSD patients with aphasia is encouraged. Moreover, HDRS-17, MADRS, and BDI scales are the most commonly used scales to evaluate PSD. Nevertheless, when used to assess PSD patients with aphasia, the ADRS scale is more appropriate.

The pathological mechanism by which tDCS improves symptoms in PSD patients with CPSP in Hassan's study is attributed to a common target of chronic pain and depression: the DLPFC, whose cortical excitation is increased due to tDCS stimulation. However, due to only one case report and short follow-up time, more experiments are needed in the future to generalize the conclusion that tDCS can improve depression and pain in PSD patients with CPSP.

Transcranial direct current stimulation is an emerging neuromodulation technique that obsesses the benefits of convenience, safety profile, and lower cost compared with TMS (49). Only a few studies have reported transient skin irritation, itching, erythema, and tingling (50). Although the studies under consideration do not report any adverse events, it is necessary to validate the safety parameters of tDCS because the intensity and location of the current may vary depending on the local anatomy and lesion time (38). Unfortunately, the study only from Valiengo in 2017 is reported to conduct a tDCS adverse effects questionnaire for assessing safety. Consequently, safety has to be proven in further high-quality research with clinical assessments. In addition to better safety, tDCS allows a reliable sham condition for a controlled study, which is difficult to easily identify from active stimulation (51). These advantages facilitate the application of tDCS in both hospitals, and especially homes for bedridden patients, boosting the generalization of tDCS (52).

There are many factors that might influence the tDCS effects. TDCS dose has been taken into consideration recently. A meta-analysis shows that tDCS dose may be an independent predictor of better efficacy (53). Evidence suggests that the effects of tDCS are the results of cumulative effects (54). This phenomenon has been well confirmed in the study of Hassan et al., with better scores in BDI after increased tDCS dose. However, among the included studies, authors conducted that 20 is the longest session owing to a low rate of back to the clinical center. Therefore, patients who have trouble returning to the clinical center (e.g., physical disabilities, living in a remote area, and so on) are in pressing need of an alternative approach that they can use when they stay away from the clinic or research facility. The good news is that a comprehensive guide to operating tDCS safely in home settings and clinical use is provided recently. The guideline can facilitate further clinical research to a certain extent (54).

Another factor affecting the tDCS effects is stimulation protocols. For example, four studies (40–43) recorded the stimulation parameters were 2 mA for 30 min daily. However, two studies (44, 45) reported patients receiving sessions of anodal stimulations of 2 mA intensity for only 20 min. Moreover, in the sham/controlled group, a brief stimulation period, 60 s (43) or 30s (40), is conducted to mimic common skin effects experienced just after stimulation for 30 min, followed by no stimulation during the remaining period. While in the study of anodal stimulations of 2 mA for only 20 min, stimulation is stopped after 15 s in the control group. Consequently, an optimal stimulation protocol is needed to formulate. In addition, factors regarding the PSD such as stroke types, time since stroke, and the duration of depression onset after stroke also need to be considered in the tDCS effects. Homogeneity tests, therefore, need to be conducted before each study, as shown in studies by An et al., Li et al., and Valiengo et al. (43).

FNIRS is a promising noninvasive neuroimaging technique used to measure activation-induced changes in the cerebral hemoglobin concentrations of oxyhemoglobin (ΔHbO) and deoxyhemoglobin (ΔHbR) (55). Greater blood flow can be detected by oxyhemoglobin in veins of activation of brain cortical neurons than inactive ones (56). In Li's study, the authors use fNIRS before and after treatment, and reaction times and relative concentration changes of oxyhemoglobin (Oxy-Hb) in the PFC are assessed using an emotional face sex judgment task and a “1-back” working memory task. They conclude that enhancing aerobic metabolism in the PFC is the main mechanism of tDCS in improving the processing of negative emotions and working memory in patients with PSD. In comparison with the existing neuroimaging techniques involving direct neural activation measurement methods such as functional magnetic resonance imaging (fMRI) and electroencephalography (EEG), fNIRS is more attractive with the advantage of higher spatial resolution and lower susceptibility to the movement artifact (55). Thus, fNIRS technology can be applied in the future to explore the neural mechanism of tDCS improving PSD and even other post-stroke diseases.

Two studies (41, 44) report that antidepressants are used as a concomitant task, and one study records that conventional occupational therapy is conducted during tDCS stimulation. Furthermore, in Bueno's study, although fluoxetine alone did not improve depression in the patient, the combined use of fluoxetine with tDCS led to mood and affective improvement. The American Heart Association/American Stroke Association (AHA/ASA) recommends the pharmacological treatment of PSD with selective serotonin reuptake inhibitors (SSRIs) or tricyclic antidepressants (TCAs), especially for patients in rehabilitation settings (7). According to a factorial, randomized, and controlled trial, tDCS stimulation combines with sertraline increases the efficacy of each treatment (57). Therefore, a combination of tDCS stimulation with other therapies may lead to better clinical outcomes compared with mono-therapy.

Another interesting finding is that it takes a long time for tDCS to reach its maximum effect. This was well-demonstrated in Valiengo et al.'s (42, 43) and Hassan's experiments. As has been discussed earlier, tDCS also has long-lasting aftereffects, which involve a lot of neurotransmitters and pathological processes in the central nervous system, and its effects are associated with synaptic plasticity changes in the brain (50). The findings could guide clinical trials in which tDCS should be used before concomitant therapies to help maximize its aftereffects when more than two interventions are needed to treat the diseases.

Limitations were analyzed in five studies except for Bueno's study. An's and Valiengo's studies had a common limitation, a small sample size. In addition to this, as we all know, RCTs are the most convincing designs to assess the efficacy of new treatments or interventions (58). However, there is very low-certainty evidence from these conclusions because only one RCT research is included. As a result, we can only put forward a weak recommendation in favor of the tDCS for PSD. Therefore, future multi-center, large-sample clinical trials are needed to allow generalization of the results. Furthermore, follow-up of depression and improvement in quality of life in patients with PSD is necessary.

## Conclusion

Based on the current evidence, tDCS presents promising results for the treatment of PSD. Moreover, tDCS is also effective in PSD patients with aphasia or CPSP. However, an optimal stimulation protocol is needed to formulate. Thus, the development of robustly controlled, randomized, and high-quality clinical trials to further assess the utility of tDCS as a therapeutic tool for the treatment of PSD survivors is encouraged.

## Data availability statement

The original contributions presented in the study are included in the article/Supplementary material, further inquiries can be directed to the corresponding author.

## Author contributions

WH and YN managed the literature searches and analyses and wrote the first draft of the manuscript. YL reviewed and revised the manuscript. WH and XG carried out the data extraction and all the authors were assigned to assess methodological quality. YG analyzed the data and made tables. When it came to any disagreements, YL was consulted to cope with inconsistencies. All authors contributed to and approved the final manuscript.
