# Supplementary material for: Transcranial direct current stimulation for the treatment of post-stroke depression: A systematic review
Source: Front Neurol. 2023 Jan 18;13:955209. doi: 10.3389/fneur.2022.955209 (PMC9893893; doi:10.3389/fneur.2022.955209)
Supplement: Supplementary file 1 [file Table_1.docx]

**Supplementary Information**

Search string used to identify relevant literature within the Pubmed database：

| **Search number** | **Search terms** | **Results** |
| --- | --- | --- |
| #1 | "Stroke"[Mesh] | 157,763 |
| #2 | (stroke[Title/Abstract]) OR (((((((((((((((((((((((((((Cerebrovascular Accident[Title/Abstract]) OR (Cerebrovascular Accidents[Title/Abstract])) OR (CVA (Cerebrovascular Accident[Title/Abstract]))) OR (CVAs (Cerebrovascular Accident[Title/Abstract]))) OR (Cerebrovascular Apoplexy[Title/Abstract])) OR (Stroke, Cerebrovascular[Title/Abstract])) OR (Apoplexy, Cerebrovascular[Title/Abstract])) OR (Vascular Accident, Brain[Title/Abstract])) OR (Brain Vascular Accident[Title/Abstract])) OR (Brain Vascular Accidents[Title/Abstract])) OR (Vascular Accidents, Brain[Title/Abstract])) OR (Cerebrovascular Stroke[Title/Abstract])) OR (Cerebrovascular Strokes[Title/Abstract])) OR (Strokes, Cerebrovascular[Title/Abstract])) OR (Apoplexy[Title/Abstract])) OR (Cerebral Stroke[Title/Abstract])) OR (Cerebral Strokes[Title/Abstract])) OR (Stroke, Cerebral[Title/Abstract])) OR (Strokes, Cerebral[Title/Abstract])) OR (Stroke, Acute[Title/Abstract])) OR (Acute Stroke[Title/Abstract])) OR (Acute Strokes[Title/Abstract])) OR (Strokes, Acute[Title/Abstract])) OR (Cerebrovascular Accident, Acute[Title/Abstract])) OR (Acute Cerebrovascular Accident[Title/Abstract])) OR (Acute Cerebrovascular Accidents[Title/Abstract])) OR (Cerebrovascular Accidents, Acute[Title/Abstract])) | 291,100 |
| #3 | #1 OR #2 | 329,076 |
| #4 | "Depression"[Mesh] | 139,700 |
| #5 | ((((((depression[Title/Abstract]) OR (Depressive Symptoms[Title/Abstract])) OR (Depressive Symptom[Title/Abstract])) OR (Symptom, Depressive[Title/Abstract])) OR (Symptoms, Depressive[Title/Abstract])) OR (Emotional Depression[Title/Abstract])) OR (Depression, Emotional[Title/Abstract]) | 407,541 |
| #6 | #4 OR #5 | 437,209 |
| #7 | "Transcranial Direct Current Stimulation"[Mesh] | 4,132 |
| #8 | (transcranial direct current stimulation[Title/Abstract]) OR ((((((((((((((((((((((((tDCS[Title/Abstract]) OR (Cathodal Stimulation Transcranial Direct Current Stimulation[Title/Abstract])) OR (Cathodal Stimulation tDCS[Title/Abstract])) OR (Cathodal Stimulation tDCSs[Title/Abstract])) OR (Stimulation tDCS, Cathodal[Title/Abstract])) OR (Stimulation tDCSs, Cathodal[Title/Abstract])) OR (tDCS, Cathodal Stimulation[Title/Abstract])) OR (tDCSs, Cathodal Stimulation[Title/Abstract])) OR (Transcranial Random Noise Stimulation[Title/Abstract])) OR (Transcranial Alternating Current Stimulation[Title/Abstract])) OR (Transcranial Electrical Stimulation[Title/Abstract])) OR (Electrical Stimulation, Transcranial[Title/Abstract])) OR (Electrical Stimulations, Transcranial[Title/Abstract])) OR (Stimulation, Transcranial Electrical[Title/Abstract])) OR (Stimulations, Transcranial Electrical[Title/Abstract])) OR (Transcranial Electrical Stimulations[Title/Abstract])) OR (Anodal Stimulation Transcranial Direct Current Stimulation[Title/Abstract])) OR (Anodal Stimulation tDCS[Title/Abstract])) OR (Anodal Stimulation tDCSs[Title/Abstract])) OR (Stimulation tDCS, Anodal[Title/Abstract])) OR (Stimulation tDCSs, Anodal[Title/Abstract])) OR (tDCS, Anodal Stimulation[Title/Abstract])) OR (tDCSs, Anodal Stimulation[Title/Abstract])) OR (Repetitive Transcranial Electrical Stimulation[Title/Abstract])) | 7,651 |
| #9 | #7 OR #8 | 8,040 |
| #10 | #3 AND #6 AND #9 | 45 |

Search string used to identify relevant literature within the Cochrane database：

| **ID** | **Search** | **Hits** |
| --- | --- | --- |
| #1 | MeSH descriptor: [Stroke] explode all trees | 11217 |
| #2 | (Cerebrovascular Accident):ti,ab,kw OR (Cerebrovascular Accidents):ti,ab,kw OR (CVA (Cerebrovascular Accident)):ti,ab,kw OR (CVAs (Cerebrovascular Accident)):ti,ab,kw OR (Cerebrovascular Apoplexy):ti,ab,kw OR (Apoplexy, Cerebrovascular):ti,ab,kw OR (Vascular Accident, Brain):ti,ab,kw OR (Brain Vascular Accident):ti,ab,kw OR (Brain Vascular Accidents):ti,ab,kw OR (Vascular Accidents, Brain):ti,ab,kw OR (Cerebrovascular Stroke):ti,ab,kw OR (Cerebrovascular Strokes):ti,ab,kw OR (Stroke, Cerebrovascular):ti,ab,kw OR (Strokes, Cerebrovascular):ti,ab,kw OR (Apoplexy):ti,ab,kw OR (Cerebral Stroke):ti,ab,kw OR (Cerebral Strokes):ti,ab,kw OR (Stroke, Cerebral):ti,ab,kw OR (Strokes, Cerebral):ti,ab,kw OR (Stroke, Acute):ti,ab,kw OR (Acute Stroke):ti,ab,kw OR (Acute Strokes):ti,ab,kw OR (Strokes, Acute):ti,ab,kw OR (Cerebrovascular Accident, Acute):ti,ab,kw OR (Acute Cerebrovascular Accident):ti,ab,kw OR (Acute Cerebrovascular Accidents):ti,ab,kw OR (Cerebrovascular Accidents, Acute):ti,ab,kw | 32486 |
| #3 | #1 OR #2 | 38270 |
| #4 | MeSH descriptor: [Depression] explode all trees | 13789 |
| #5 | (Depressive Symptoms):ti,ab,kw OR (Depressive Symptom):ti,ab,kw OR (Symptom, Depressive):ti,ab,kw OR (Symptoms, Depressive):ti,ab,kw OR (Emotional Depression):ti,ab,kw OR (Depression, Emotional):ti,ab,kw | 22766 |
| #6 | #4 OR #5 | 31227 |
| #7 | MeSH descriptor: [Transcranial Direct Current Stimulation] explode all trees | 964 |
| #8 | (tDCS):ti,ab,kw OR (Cathodal Stimulation Transcranial Direct Current Stimulation):ti,ab,kw OR (Cathodal Stimulation tDCS):ti,ab,kw OR (Cathodal Stimulation tDCSs):ti,ab,kw OR (Stimulation tDCS, Cathodal):ti,ab,kw OR (Stimulation tDCSs, Cathodal):ti,ab,kw OR (tDCS, Cathodal Stimulation):ti,ab,kw OR (tDCSs, Cathodal Stimulation):ti,ab,kw OR (Transcranial Random Noise Stimulation):ti,ab,kw OR (Transcranial Alternating Current Stimulation):ti,ab,kw OR (Transcranial Electrical Stimulation):ti,ab,kw OR (Electrical Stimulation, Transcranial):ti,ab,kw OR (Electrical Stimulations, Transcranial):ti,ab,kw OR (Stimulation, Transcranial Electrical):ti,ab,kw OR (Stimulations, Transcranial Electrical):ti,ab,kw OR (Transcranial Electrical Stimulations):ti,ab,kw OR (Anodal Stimulation Transcranial Direct Current Stimulation):ti,ab,kw OR (Anodal Stimulation tDCS):ti,ab,kw OR (Anodal Stimulation tDCSs):ti,ab,kw OR (Stimulation tDCS, Anodal):ti,ab,kw OR (Stimulation tDCSs, Anodal):ti,ab,kw OR (tDCS, Anodal Stimulation):ti,ab,kw OR (tDCSs, Anodal Stimulation):ti,ab,kw OR (Repetitive Transcranial Electrical Stimulation):ti,ab,kw | 5112 |
| #9 | #7 OR #8 | 5180 |
| #10 | #3 AND #6 AND #9 | 4 |
